# Temporal trends in respiratory mortality and short-term effects of air pollutants in Shenyang, China

**DOI:** 10.1007/s11356-018-1270-5

**Published:** 2018-02-09

**Authors:** Xiaoxia Xue, Jianping Chen, Baijun Sun, Baosen Zhou, Xuelian Li

**Affiliations:** 1 Science Experiment Center, China Medical University, No.77 Puhe Road, Shenyang North New Area, Shenyang, 110122 Liaoning Province People’s Republic of China; 2 Shenyang Center for Disease Control and Prevention, No.37 Qishan Road, Huanggu District, Shenyang, 110031 Liaoning Province People’s Republic of China; 3 Department of Epidemiology, School of Public Health, China Medical University, No.77 Puhe Road, Shenyang North New Area, Shenyang, 110122 Liaoning Province People’s Republic of China

**Keywords:** Air pollution, Mortality, Respiratory disease, Lung cancer, Case-crossover, Particulate matter, Population aging

## Abstract

Short-term exposures to air pollution are associated with acute effects on respiratory health. This study aimed to describe 10-year temporal trends in respiratory mortality in the urban areas of Shenyang, China, according to gender and age and estimate the effects of air pollution on respiratory diseases (ICD-10J00-J99) and lung cancer (ICD-10 C33-C34) using a case-crossover design. During the study period 2013–2015, the exposure-response relationship between ambient air pollutants and mortality data was fitted by a quasi-Poisson model. Age-standardized mortality rates for a combined number of respiratory diseases and for lung cancer declined in Shenyang; however, death counts increased with aging. Deaths from respiratory diseases increased by 4.7% (95% CI, 0.00–9.9), and lung cancer mortality increased by 6.5% (95% CI, 1.2–12.0), both associated with a 10 μg/m^3^ increase in exposure to particulate matter < 2.5 μg in diameter (PM2.5). Moreover, males in Shenyang’s urban areas were more susceptible to the acute effects of PM2.5 and SO_2_ exposure; people aged ≥ 65 years had a high susceptibility to ozone, and those aged < 65 years were more susceptible to other air pollutants. These results provided an updated estimate of the short-term effects of air pollution in Shenyang. Since population aging is also associated with increasing mortality from respiratory diseases and lung cancer, reinforcing air quality control measures and health-promoting behaviors is urgent and necessary in Shenyang.

**Electronic supplementary material:**

The online version of this article (10.1007/s11356-018-1270-5) contains supplementary material, which is available to authorized users.

## Introduction

Over the recent decades, the air quality in China has worsened due to rapid economic development, accelerated urbanization, and industrialization (Zhang and Cao 2015). China now ranks as one of the top polluted countries in the world. Disability-adjusted life years attributed to outdoor particulate matter (PM) exposure has been increasing in China over the past years (Guan et al. 2016). Epidemiological studies indicated that patients with chronic obstructive pulmonary disease are at an increased risk of death associated with the exposure to particle air pollutants (Sunyer et al. 2000). Previous evidences indicated that ambient air pollutants have long-term and short-term adverse effects on mortality and morbidity for cardiopulmonary diseases in developing countries, especially in China (Li et al. 2017). Several reviews of the adverse health effects of air pollution in the Chinese population yield similar results (Lu et al. 2015; Shang et al. 2013). In China, the short-term effect of the air pollution on the health burden has been studied in several cities such as Beijing, Shanghai, Wuhan, and Shenzhen (Lai and Brimblecombe 2017; Li et al. 2015; Liang et al. 2017; Lin et al. 2017; Zhang et al. 2017). A systematic review of 33 time series and case-crossover studies provided additional insights into the heterogeneity in effects on daily mortality after exposure to air pollution in China (Shang et al. 2013). The combined estimates in this meta-analysis indicated that exposures to all pollutants of interest significantly enhanced the risks of mortality in Chinese population, and the effects demonstrated heterogeneity in relation to the air pollution sources and the chemical composition of ambient particle.

Because of the regional or seasonal heterogeneity, the urban air in northern China was generally more polluted than that in the south mainly due to the coal smoke. Shenyang is an old industrial city in Northeast China. Since the winter is almost a half-year long, the use of coal-burning heating systems is very common in the city (Geng et al. 2013). Haze or smog episodes over the urban areas have become a common feature of winter, with levels of PM2.5 frequently exceeding 500 μg/m^3^. Associations between exposure to air pollution and mortality were observed in epidemiological studies in Shenyang (Ma et al. 2011; Zhang et al. 2011). However, updated air pollution epidemiological study on all pollutants of interest, especially on PM2.5 in Shenyang city, is still very limited. Meanwhile, rapid population aging is occurring in Shenyang city. The fraction of population aged ≥ 65 years in Shenyang’s urban areas has increased by 13% since 2013, compared with only 9% in 2005. Increases in natural mortality from PM exposure have been found among susceptible subgroups who were elderly persons (aged ≥ 65 years) and with chronic morbidity (Alessandrini et al. 2016). To better understand the association between air pollutants and mortality in Shenyang, especially in different age or gender groups, we evaluated 10-year temporal changes in respiratory mortality and lung cancer mortality, and also we examined the association between air pollutant exposure and daily respiratory death in Shenyang from 2013 to 2015 using a case-crossover analysis.

## Materials and methods

### Setting

Shenyang had an urban population of 3.8 million in 2014. The city typically has a sub-humid temperate continental climate, with an average temperature of 8 °C (46 °F). Its summer is not very hot, and the hottest month is July, with an average temperature of 23 °C (73 °F). The winter is cold and dry, with the coldest month being January, presenting an average temperature of − 16 °C (3 °F). Being one of the old cradles of industry in China, Shenyang is experiencing serious air pollution problems in recent years.

### Data collection

Data regarding average daily air mass index (Air Quality Index, AQI) in Shenyang were obtained from the China National Environmental Monitoring Centre from January 2013 to December 2015. Since AQI was not routinely monitored in early 2013, data from the Mission China air quality monitoring program of the USA were collected. The 13 monitoring stations established in Shenyang were fully automated and routinely monitored levels of six criteria pollutants, including particulate matter < 2.5 μm in diameter (PM2.5), PM < 10 μm in aerodynamic diameter (PM10), sulfur dioxide (SO_2_), nitrogen dioxide (NO_2_), carbon monoxide (CO), and ozone (O_3_). The daily average concentration of pollutants at these urban stations was represented by the mean of the daily average concentrations of atmospheric pollutants of 13 national fixed-site stations, converted from the concentrations of multiple pollutants based on the Ambient Air Quality Standard (GB 3095-2012).

Daily meteorological data were obtained for the same period from the China Meteorological Administration from 1 January 2013 to 31 December 2015, including information on daily average temperature, barometric pressure, and relative humidity.

Daily mortality data of residents living in the urban areas of Shenyang were collected from the death registration system of Shenyang Center for Disease Control and Prevention during the same period. Causes of deaths were coded according to the International Classification of Diseases, 10th Revision (ICD-10); respiratory deaths were classified under codes J00-J99, and lung cancer under codes C33-C34. In this study, death counts for a combined number of respiratory diseases (J00-J99), lung cancer (C33-C34), and three common respiratory diseases (chronic lower respiratory disease [CLRD, J40-J47], pneumonia [J18], and pneumoconiosis [J61-J62]) were analyzed.

### Data analysis

Mortality rates were age-standardized using the direct method based on the Chinese standard population of 2010. The average annual percentage of change (AAPC) and its 95% confidence interval (CI) were calculated using joinpoint regression analysis (Kim et al. 2000).

A time-stratified case-crossover design was used to evaluate the associations between the daily mean concentration of pollutants and the daily mortality count of each outcome, with adjustment for same-day meteorological factors including daily average temperature and relative humidity introduced as concomitant variables and considering the lag effects of air pollutant increments. Control days were chosen such that cases and controls were matched on the calendar month and day of the week. Air pollutant concentrations were examined in single-pollutant models for each disease category and the effects of lagging exposure for 0, 1, and 2 days (lag 0, lag l, and lag 2 days, respectively) as well as cumulative lags (lag 01 and lag 02) were assessed. Multiple-pollutant models (in which three pollutants of PM2.5, SO_2_ and NO_2_ were included together) examined the independence of any significant or marginally significant (*P* values ≈0.05) associations observed in single-pollutant models. PM10 and CO were excluded from the multiple-pollutant models due to their high correlation with other pollutants. For each pollutant, odds ratios (ORs) and 95% CIs were calculated using the Poisson regression. The regression model was modified by a quasi-Poisson model, which accounted for over dispersion in utilizing the GLM function of R3.3.2 in the stats package.

## Results

### Data description

The demographic characteristics of the study population for respiratory deaths are shown in Table 1. From 1 January 2005 to 31 December 2015, a total of 29,693 respiratory deaths and 29,112 lung cancer deaths were recorded. Pneumonia and CLRD accounted for 79.08% (23,482) of the total number of respiratory deaths.

**Table 1:** Demographic characteristics of the study population

Characteristics		Respiratory diseases (J00-J99)	Pneumonia (J18)	CLRD (J40-J47)	Pneumoconiosis (J61-J62)	Lung cancer (C33-C34)
Gender	Male deaths (%)	16,402 (55.24%)	7465 (58.90%)	5325 (49.26%)	252 (87.20%)	17,656 (60.65%)
	Female deaths (%)	13,291 (44.76%)	5208 (41.10%)	5484 (50.74%)	37 (12.80%)	11,456 (39.35%)
Age	Mean ± SD	78.14 ± 11.86	78.72 ± 12.53	78.30 ± 10.30	80.24 ± 12.28	70.87 ± 11.60

*CLRD* chronic lower respiratory disease

Death rates were age-standardized based on the Chinese standard population of the Sixth National Population Census. The age-standardized mortality rates (per 100,000 per year) of a combined number of respiratory diseases decreased from 82.87 in 2005 to 37.05 in 2015 (Table 2). Respiratory mortality has decreased significantly since 2006 by − 5.1 (95% CI, − 7.2 to − 3.0) per 100,000 per year in men and by − 8.1 (95% CI, − 10.6 to − 5.6) per 100,000 per year in women. The AAPC from 2005 to 2015 also indicates a continued decrease in lung cancer mortality (male, − 4.2 [95% CI, − 7.2 to − 3.0]; female, − 5.7 [95% CI, − 7.4 to − 4.0]).

**Table 2:** Respiratory disease and lung cancer mortality in the urban areas of Shenyang, China, in 2005–2015 (per 100,000 per year)

		Respiratory diseases (J00-J99)		Pneumonia (J18)			CLRD (J40-J47)				Pneumoconiosis (J61-J62)			Lung cancer (C33-C34)		
	Year	Count	Crude rate	ASR	Count	Crude rate	ASR	Count	Crude rate	ASR	Count	Crude rate	ASR	Count	Crude rate	ASR
	2005	2428	70.16	82.87	638	18.44	22.14	1225	35.40	41.21	39	1.13	1.14	2304	66.58	65.97
	2006	2216	63.78	57.17	558	16.06	14.78	1018	29.30	26.02	27	0.78	0.65	2386	68.67	56.33
	2007	2305	65.92	63.01	577	16.50	16.12	1021	29.20	27.39	21	0.60	0.54	2450	70.07	58.70
Total	2008	2479	70.68	58.82	900	25.65	21.76	958	27.30	22.36	14	0.40	0.30	2561	72.99	56.19
	2009	2461	69.91	57.22	995	28.26	23.51	912	25.91	20.85	24	0.68	0.50	2482	70.51	54.52
	2010	2686	73.79	44.31	1171	32.17	19.47	1107	30.41	18.02	28	0.77	0.43	2769	76.07	48.16
	2011	2654	70.57	39.13	1144	30.42	16.89	1046	27.81	15.26	24	0.64	0.33	2742	72.91	43.77
	2012	3021	79.93	42.22	1402	37.09	19.49	1051	27.81	14.54	38	1.01	0.51	2929	77.50	45.49
	2013	2957	78.58	39.96	1528	40.60	20.48	758	20.14	9.97	32	0.85	0.39	2598	69.04	38.63
	2014	3362	88.35	42.69	2164	56.89	27.50	881	23.14	10.96	25	0.66	0.29	2995	78.66	43.00
	2015	3124	81.42	37.05	1596	41.60	18.89	777	20.25	8.86	17	0.44	0.19	2896	75.51	39.69
AAPC, 95% CI		− 6.6 [− 8.8, − 4.3]			1.9 [− 2.2, 6.2]			− 13.1 [− 15.0, − 11.1]			− 11.2 [− 16.5, − 5.5]			− 6.6 [− 8.8, − 4.3]		
	2005	1268	73.64	21.89	377	33.92	28.52	584	34.11	42.28	35	2.03	2.22	1345	78.11	82.61
	2006	1216	70.40	18.64	322	30.17	18.11	521	30.32	28.52	24	1.39	1.26	1469	85.05	74.50
	2007	1235	71.20	19.37	336	28.59	20.45	496	28.79	28.27	18	1.04	0.97	1528	88.09	78.05
	2008	1336	76.95	31.04	539	26.49	28.02	460	26.67	23.17	14	0.81	0.65	1565	90.14	74.38
Male	2009	1358	78.29	33.50	581	25.31	29.59	439	25.48	22.08	19	1.10	0.84	1465	84.46	70.17
	2010	1474	82.24	38.67	693	30.19	25.4	541	30.41	19.47	24	1.34	0.81	1625	90.67	62.14
	2011	1502	81.19	38.11	705	28.38	23.08	525	28.60	16.98	21	1.14	0.63	1661	89.78	58.67
	2012	1698	91.42	45.87	852	27.73	26.39	515	27.98	16.13	34	1.83	1.03	1752	94.33	60.04
	2013	1684	91.16	49.75	919	19.92	27.76	368	20.09	11.00	26	1.41	0.70	1624	87.91	53.54
	2014	1872	100.30	66.99	1250	23.03	36.18	430	23.28	12.33	23	1.23	0.60	1808	96.82	57.54
	2015	1759	93.95	47.59	891	22.00	24.04	412	22.30	11.05	14	0.75	0.35	1815	96.94	55.36
AAPC, 95% CI		− 5.1 [− 7.2, − 3.0]			2.4 [− 1.7, 6.6]			− 11.9 [− 13.7, − 11.0]			− 11.0 [− 16.5, − 5.1]			− 4.2 [− 7.2, − 3.0]		
	2005	1160	66.72	15.01	261	36.87	16.93	641	37.06	35.22	4	0.23	0.17	959	55.16	51.25
	2006	1000	57.23	13.51	236	28.44	11.91	497	28.58	30.23	3	0.17	0.12	917	52.48	39.92
	2007	1070	60.73	13.68	241	29.80	12.61	525	29.99	25.94	3	0.17	0.16	922	52.33	41.31
	2008	1143	64.55	20.37	361	28.10	16.37	498	28.27	22.27	0	0.00	0.00	996	56.20	40.19
Female	2009	1103	61.77	23.18	414	26.49	18.20	473	26.65	19.11	5	0.28	0.19	1017	56.95	40.71
	2010	1212	65.60	25.87	478	30.63	14.40	566	30.84	16.40	4	0.22	0.11	1144	61.91	35.57
	2011	1152	60.29	22.97	439	27.27	11.74	521	27.46	14.08	3	0.16	0.08	1081	56.57	30.73
	2012	1323	68.83	28.61	550	27.88	13.81	536	28.11	12.08	4	0.21	0.10	1177	61.23	32.75
	2013	1273	66.44	31.79	609	20.36	14.41	390	20.51	10.37	6	0.31	0.14	974	50.84	25.47
	2014	1490	76.85	47.16	914	23.25	20.32	451	23.47	8.90	2	0.10	0.04	1187	61.18	30.51
	2015	1365	69.49	35.89	705	18.58	14.75	365	19.00	7.64	3	0.15	0.06	1082	55.08	26.59
AAPC, 95% CI		−8.1 [−10.6, −5.6]			1.5 [−2.7, 5.9]			−14.2 [−16.2, −12.1]			–			−5.7 [−7.4, −4.0]		

*CLRD* chronic lower respiratory disease, *ASR* age-standardized rate, per 100,000 per year, *AAPC* average annual percentage of change, *CI* confidence interval

Despite decreases in age-standardized death rates, the absolute number of respiratory deaths, occurring more frequently at older ages, continues to increase. The death counts for a combined number of respiratory diseases increased by 29% between 2005 and 2015 and were mainly associated with population aging. The number of deaths from pneumonia increased more than twofold during the same period, but a non-significantly increased age-standardized rate was found (1.9; 95% CI, − 2.2 to 6.2).

From 2013 to 2015, a daily average of 8.54 persons died from respiratory diseases and 7.67 from lung cancer in the urban areas of Shenyang (Table 3). During the study period, the average concentration of PM2.5 and PM10 was 70.71 and 117.08 μg/m^3^, respectively.

**Table 3:** Mortality, air pollution, and meteorological measurements in Shenyang, China, 2013–2015

	Mean	SD	IQR	Min	Q1	Q2	Q3	Max
PM2.5 (μg/m^3^)	70.71	60.36	53.00	10.00	34.00	53.00	87.00	848.00
PM10 (μg/m^3^)	117.08	76.94	77.00	19.00	69.00	97.00	146.00	912.00
SO_2_ (μg/m^3^)	68.45	69.50	81.00	3.00	19.00	38.00	100.00	379.00
NO_2_ (μg/m^3^)	44.63	18.01	24.00	10.00	31.00	41.00	55.00	125.00
CO (mg/m^3^)	1.18	0.62	0.62	0.32	0.73	0.99	1.35	4.93
O_3_ (μg/m^3^)	56.19	30.77	46.00	6.00	31.00	53.00	77.00	178.00
Mean temperature (°C)	8.91	13.17	24.00	− 21.00	− 3.00	11.00	21.00	29.00
Mean humidity (%)	61.75	15.65	22.5	21.00	51.00	63.00	73.50	95.00
Respiratory disease deaths (J00-J99)	8.54	3.17	4	1	6	8	10	21
Lung cancer deaths (C33-C34)	7.67	2.94	5	0	5	8	10	20

*SD* standard deviation, *IQR* interquartile range, *Min* minimum, *Q* quartile, *Max* maximum, *PM2.5* particulate matter < 2.5 μm in diameter, *PM10* particulate matter < 10 μm in diameter

Spearman’s rank correlation coefficients between pollutants and weather variables are presented in Table 4. All five pollutants (PM2.5, PM10, SO_2_, NO_2_, CO) were highly correlated, especially between PM2.5 and PM10 (*r* = 0.915); however, they were all negatively correlated with ozone. Furthermore, the daily mean temperature is negatively correlated with air pollutants except for ozone; the largest correlation coefficient was found in SO_2_ with − 0.767.

**Table 4:** Spearman’s rank correlation coefficients between pollutants and weather variables

	PM2.5	PM10	SO_2_	NO_2_	CO	O_3_	Mean humidity	Mean temperature
PM2.5 (μg/m^3^)	1.000							
PM10 (μg/m^3^)	0.915*	1.000						
SO_2_ (μg/m^3^)	0.671*	0.648*	1.000					
NO_2_ (μg/m^3^)	0.666*	0.610*	0.585*	1.000				
CO (mg/m^3^)	0.828*	0.777*	0.673*	0.595*	1.000			
O_3_ (μg/m^3^)	− 0.256*	− 0.208*	− 0.555*	− 0.514*	− 0.253*	1.000		
Mean humidity (%)	0.037	− 0.135*	− 0.182*	− 0.039	0.129*	− 0.052	1.000	
Mean temperature (°C)	− 0.362*	− 0.334*	− 0.767*	− 0.320*	− 0.322*	0.690*	0.222*	1.000

*P < 0.01

### Associations between air pollutants and respiratory deaths

The effect estimates of each air pollutant on daily respiratory mortality after controlling for meteorological and seasonal influences are shown in Table 5. Increased mortality was observed to be associated with elevated concentrations of PM2.5 on the same day. A 10 μg/m^3^ increment of PM2.5 was associated with a 4.5% increase in daily mortality for respiratory diseases and lung cancer (95% CI, 1.1–8.1%). The 10 μg/m^3^ increment of PM10 and and 1 mg/m^3^ increment of CO were associated with a 4.8% (95% CI, 0.7–9.2%) and a 1.9% (95% CI, 0.3–3.7%) increase in overall deaths, respectively. Moreover, with a 24-h lag period, associations between mortality and PM2.5 or CO levels still existed, with excess risks of 3.6% (95% CI, 0.1–7.2%) and 2.1% (95% CI, 0.4–3.8%), respectively. In addition, increments in SO_2_ exposure with a 1-day lag were associated with overall respiratory mortality, with an OR of 5.2% (1.3%, 9.3%). A 10 μg/m^3^ increase in 2-day mean PM2.5, PM10, and SO_2_ concentrations was associated with a 4.7% (95% CI, 0.5–9.0%), 5.1% (95% CI, 0.0–10.4%), and 5.9% [95% CI, 1.1–10.9%) increased risk of overall respiratory death, respectively. Same-day PM2.5 exposure or 2-day mean PM2.5 concentrations were also statistically significantly associated with lung cancer mortality, with 6.5% (95% CI, 1.2–12.0%) and 6.8% (95% CI, 0.5–13.6%) increases in mortality. For death counts attributed to respiratory diseases, CO exposure with a 1-day lag showed a significant association, with an elevated OR of 3.8% (95% CI, 1.3–6.3%) per 1 mg/m^3^ increase in CO concentration. PM2.5 exposure has a positive but weak association with respiratory diseases mortality (4.7%, 95% CI, 0–9.9%). Lung cancer mortality was also associated with PM10 and SO_2_ with 7.3 and 8.1% for an increase of 10 μg/m^3^. It was affected by PM10 level on the day of death, whereas more affected by the average level of the day and previous day’s SO_2_ concentration. For multiple-pollutant (adjusted for SO_2_ and NO_2_) models, increased mortality of lung cancer and the overall respiratory mortality were significantly associated with PM2.5, with the ORs for every 10 μg/m^3^ increase in PM2.5 being 1.087 (95% CI, 1.008–1.172) and 1.067 (95% CI, 1.015–1.122), respectively, at lag 0 day in multiple-pollutant model. SO_2_ also had certain effects on overall respiratory mortality with evident lag effects. The ORs (95% CIs) with a 10 μg/m^3^ increase in concentration of SO_2_, were 1.070 (95% CI, 1.005–1.141) and 1.084 (95% CI, 1.01–1.164) for lag 01 and lag 02, respectively. We also observed that a 10 μg/m^3^ change in NO_2_ of single-lag (lag 2) and cumulative-lag values (measured as a two or three-day average of lag 0, lag 1, and lag 2) was associated with a weak decline in daily mortality of respiratory diseases.

**Table 5:** Associations between air pollutants and mortality controlled by meteorological and seasonal influences

		Overall respiratory mortality (including lung cancer) OR (95% CI)		Respiratory disease deaths OR (95% CI)		Lung cancer deaths OR (95% CI)	
		Single-pollutant	Multiple-pollutant	Single-pollutant	Multiple-pollutant	Single-pollutant	Multiple-pollutant
PM2.5	Lag 0	*1.045 (1.011, 1.081)*	*1.067 (1.015, 1.122)*	1.028 (0.981, 1.077)	1.049 (0.978, 1.126)	*1.065 (1.012, 1.12)*	*1.087 (1.008, 1.172)*
	Lag 1	*1.036 (1.001, 1.072)*	1.021 (0.97, 1.074)	*1.047 (1.000, 1.099)*	1.054 (0.982, 1.131)	1.024 (0.973, 1.078)	0.987 (0.915, 1.064)
	Lag 2	1.006 (0.972, 1.042)	1.028 (0.977, 1.082)	0.994 (0.948, 1.043)	1.045 (0.974, 1.121)	1.021 (0.970, 1.075)	1.011 (0.936, 1.092)
	Lag 01	*1.047 (1.005, 1.090)*	1.046 (0.989, 1.105)	1.027 (0.970, 1.088)	1.046 (0.968, 1.13)	*1.068 (1.005, 1.136)*	1.046 (0.963, 1.137)
	Lag 02	1.028 (0.982, 1.076)	1.033 (0.974, 1.097)	1.002 (0.940, 1.068)	1.037 (0.954, 1.126)	1.056 (0.987, 1.131)	1.029 (0.942, 1.125)
PM10	Lag 0	*1.048 (1.007, 1.092)*		1.027 (0.970, 1.086)		*1.073 (1.009, 1.140)*	
	Lag 1	1.027 (0.986, 1.070)		1.04 (0.982, 1.101)		1.014 (0.954, 1.078)	
	Lag 2	1.001 (0.960, 1.043)		0.993 (0.937, 1.051)		1.011 (0.950, 1.076)	
	Lag 01	*1.051 (1.000, 1.104)*		1.03 (0.961, 1.104)		1.073 (0.997, 1.156)	
	Lag 02	1.037 (0.981, 1.097)		1.016 (0.940, 1.099)		1.06 (0.977, 1.151)	
SO_2_	Lag 0	1.033 (0.995, 1.073)	1.02 1(0.965, 1.079)	1.027 (0.975, 1.082)	1.042 (0.963, 1.127)	1.039 (0.983, 1.099)	0.998 (0.919, 1.085)
	Lag 1	*1.052 (1.013, 1.093)*	*1.06 (1.002, 1.122)*	1.052 (0.998, 1.109)	1.061 (0.980, 1.147)	1.052 (0.994, 1.113)	1.06 (0.975, 1.153)
	Lag 2	1.002 (0.964, 1.041)	1.015 (0.959, 1.074)	0.982 (0.931, 1.035)	1.012 (0.936, 1.095)	1.025 (0.968, 1.085)	1.018 (0.936, 1.107)
	Lag 01	1.059 (1.011, 1.109)	1.070 (1.005, 1.141)	1.052 (0.994, 1.113)	1.079 (0.987, 1.179)	*1.081 (1.009, 1.158)*	1.062 (0.967, 1.167)
	Lag 02	1.051 (0.996, 1.109)	*1.084 (1.01, 1.164)*	1.025 (0.968, 1.085)	1.102 (0.998, 1.216)	1.081 (0.998, 1.171)	1.066 (0.959, 1.185)
NO_2_	Lag 0	1.017 (0.963, 1.075)	0.926 (0.851, 1.008)	0.991 (0.918, 1.070)	0.900 (0.800, 1.013)	1.047 (0.964, 1.138)	0.955 (0.841, 1.083)
	Lag 1	1.035 (0.979, 1.094)	0.953 (0.875, 1.038)	1.022 (0.946, 1.103)	0.908 (0.807, 1.022)	1.050 (0.966, 1.140)	1.005 (0.885, 1.140)
	Lag 2	0.975 (0.922, 1.032)	0.931 (0.854, 1.015)	0.931 (0.861, 1.006)	*0.874 (0.776, 0.985)*	1.028 (0.946, 1.118)	0.998 (0.877, 1.135)
	Lag 01	1.019 (0.951, 1.092)	0.912 (0.827, 1.006)	0.968 (0.879, 1.065)	*0.860 (0.749, 0.987)*	1.079 (0.973, 1.196)	0.972 (0.839, 1.126)
	Lag 02	0.987 (0.911, 1.069)	*0.888 (0.793, 0.994)*	0.907 (0.812, 1.014)	*0.802 (0.685, 0.938)*	1.081 (0.96, 1.218)	0.991 (0.837, 1.173)
CO	Lag 0	*1.019 (1.003, 1.037)*		1.02 (0.997, 1.044)		1.018 (0.994, 1.044)	
	Lag 1	*1.021 (1.004, 1.038)*		*1.038 (1.013, 1.063)*		1.005 (0.981, 1.03)	
	Lag 2	1.001 (0.985, 1.018)		0.995 (0.973, 1.018)		1.008 (0.983, 1.034)	
	Lag 01	1.028 (0.998, 1.058)		1.028 (0.987, 1.071)		1.027 (0.984, 1.072)	
	Lag 02	1.004 (0.966, 1.043)		0.987 (0.935, 1.041)		1.023 (0.965, 1.084)	
O_3_	Lag 0	1.032 (0.980, 1.087)		1.049 (0.976, 1.128)		1.013 (0.937, 1.095)	
	Lag 1	1.000 (0.949, 1.054)		1.027 (0.955, 1.104)		0.971 (0.897, 1.051)	
	Lag 2	1.029 (0.976, 1.085)		1.065 (0.989, 1.146)		0.991 (0.916, 1.073)	
	Lag 01	1.029 (0.970, 1.092)		1.060 (0.976, 1.152)		0.996 (0.911, 1.089)	
	Lag 02	1.046 (0.979, 1.118)		1.092 (0.996, 1.197)		0.998 (0.904, 1.103)	

*OR* odds ratio, *CI* confidence interval, *PM2.5* particulate matter < 2.5 μm in diameter, *PM10* particulate matter < 10 μm in diameter

Italic ORs are statistically significant (*P* < 0.05). Measurement for every 10 μg/m^3^ increment of PM2.5, PM10, SO_2_, NO_2_, and O_3_ and 1 mg/m^3^ increment of CO

**Fig. 1:**
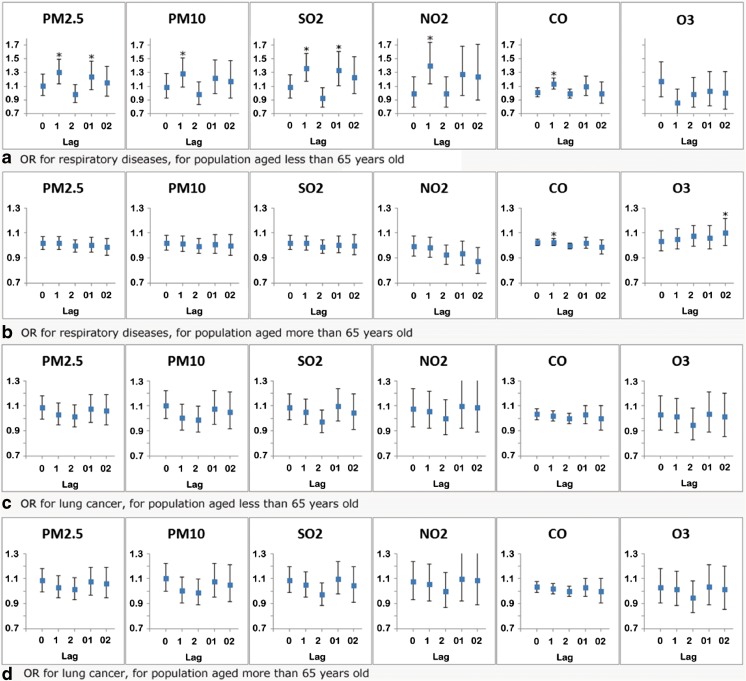
Effect estimates of air pollutants on daily mortality of lung cancer and respiratory diseases in different age groups using single-pollutant models (*P < 0.05). Measurement for every 10 μg/m^3^ increment of PM2.5, PM10, SO_2_, NO_2_, and O_3_ and 1 mg/m^3^ increment of CO

**Fig. 2:**
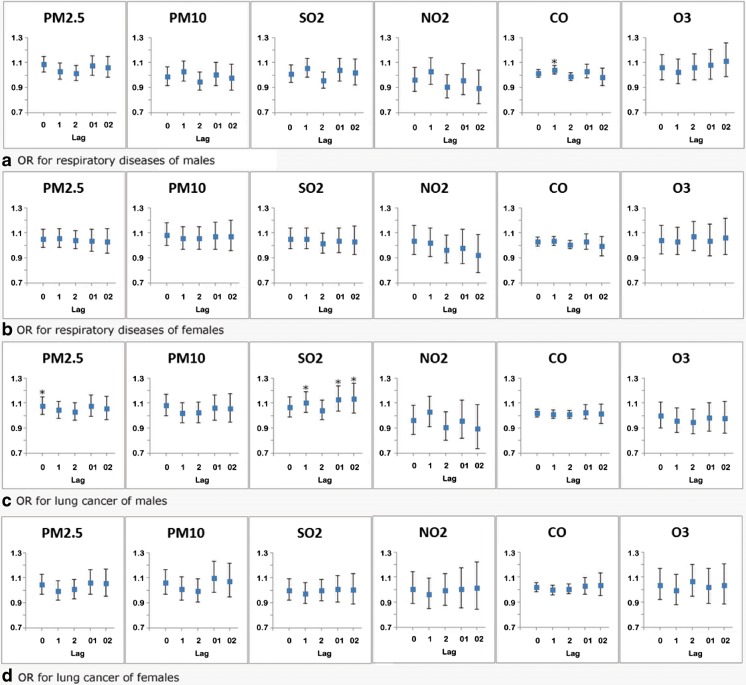
Effect estimates of air pollutants on daily mortality of lung cancer and respiratory diseases of different gender groups using single-pollutant models (*P < 0.05). Measurement for every 10 μg/m^3^ increment of PM2.5, PM10, SO_2_, NO_2_, and O_3_ and 1 mg/m^3^ increment of CO

As shown in Fig. 1 for the stratified analysis, five major air pollutants (PM2.5, PM10, SO_2_, NO_2_, and CO) significantly increased the mortality risk of respiratory diseases in persons aged < 65 years, while in elderly persons, the effects were attenuated and no longer significant except for that of CO. Increased daily mortality of respiratory diseases in persons aged over 65 years was associated with increase in ambient ozone on lag 02 (OR = 1.102, 95% CI,1.001–1.214),whereas NO_2_ level of lag 02 showed an negative association (OR = 0.873; 95% CI, 0.776–0.981). The results also showed a significant association of respiratory diseases with CO exposure in men with OR of 1.041(95% CI, 1.009–1.075) but not in women (Fig. 2).

An estimated increase of 7.7% (95% CI, 0.8–15.0%) in male lung cancer mortality was observed for a 10 μg/m^3^ increase in PM2.5 concentration on the same day, and a delayed effect of SO_2_ exposure was observed with a 10.4% (95% CI, 2.6–18.9%) increment in male lung cancer mortality in a 1-day lag. SO_2_ concentrations with a mean of 2–3 days also showed stronger effects (see Figs. 1 and 2 and Table 6, detailed data see Supplemental Material).

**Table 6:** Effect estimates of air pollutants on daily mortality of three specific respiratory diseases in single-pollutant models

		CLRD	Pneumonia	Pneumoconiosis
PM2.5	Lag 0	0.969 (0.887, 1.058)	1.022 (0.958, 1.091)	0.891 (0.688, 1.153)
	Lag 1	1.002 (0.916, 1.096)	1.044 (0.978, 1.115)	0.801 (0.625, 1.027)
	Lag 2	0.979 (0.895, 1.07)	1.005 (0.941, 1.073)	*0.709 (0.558, 0.900)*
	Lag 01	0.953 (0.856, 1.061)	1.029 (0.950, 1.113)	*0.618 (0.452, 0.845)*
	Lag 02	0.922 (0.817, 1.039)	1.019 (0.933, 1.113)	*0.561 (0.395 ,0.798)*
PM10	Lag 0	0.957 (0.861, 1.063)	1.017 (0.941, 1.100)	0.941 (0.683, 1.294)
	Lag 1	0.973 (0.873, 1.085)	1.027 (0.949, 1.112)	*0.697 (0.526, 0.924)*
	Lag 2	0.984 (0.884, 1.095)	0.982 (0.907, 1.063)	*0.745 (0.562, 0.988)*
	Lag 01	0.926 (0.812, 1.055)	1.021 (0.927, 1.124)	*0.617 (0.423, 0.900)*
	Lag 02	0.917 (0.792, 1.062)	1.010 (0.907, 1.125)	*0.612 (0.407, 0.919)*
SO_2_	Lag 0	1.018 (0.923, 1.123)	0.993 (0.924, 1.068)	1.118 (0.843, 1.484)
	Lag 1	1.024 (0.926, 1.132)	1.030 (0.958, 1.108)	0.914 (0.674, 1.239)
	Lag 2	0.990 (0.896, 1.093)	0.971 (0.903, 1.044)	0.957 (0.725, 1.263)
	Lag 01	1.019 (0.903, 1.15)	0.997 (0.912, 1.090)	0.870 (0.596, 1.271)
	Lag 02	1.000 (0.869, 1.151)	0.989 (0.890, 1.098)	0.891 (0.570, 1.392)
NO_2_	Lag 0	0.933 (0.810, 1.075)	0.988 (0.888, 1.099)	1.098 (0.721, 1.673)
	Lag 1	1.001 (0.867, 1.156)	0.993 (0.893, 1.105)	0.862 (0.556, 1.336)
	Lag 2	0.915 (0.791, 1.058)	0.930 (0.836, 1.035)	0.708 (0.340, 1.460)
	Lag 01	0.914 (0.766, 1.091)	0.950 (0.832, 1.085)	0.703 (0.410, 1.206)
	Lag 02	0.833 (0.677, 1.025)	0.903 (0.774, 1.053)	0.846 (0.233, 1.554)
CO	Lag 0	1.018 (0.974, 1.063)	1.015 (0.983, 1.047)	0.897 (0.804, 1.001)
	Lag 1	*1.058 (1.01, 1.108)*	1.032 (0.999, 1.066)	0.971 (0.86, 1.095)
	Lag 2	0.983 (0.943, 1.025)	1.019 (0.987, 1.052)	*0.839 (0.748, 0.941)*
	Lag 01	1.018 (0.941, 1.101)	1.042 (0.985, 1.103)	*0.752 (0.578, 0.978)*
	Lag 02	0.942 (0.854, 1.039)	1.042 (0.964, 1.126)	*0.691 (0.491, 0.973)*
O_3_	Lag 0	1.059 (0.924, 1.213)	1.023 (0.926, 1.130)	1.141 (0.767, 1.697)
	Lag 1	1.015 (0.886, 1.163)	0.991 (0.897, 1.096)	*1.505 (1.020, 2.220)*
	Lag 2	1.123 (0.976, 1.292)	1.058 (0.956, 1.171)	1.040 (0.719, 1.504)
	Lag 01	1.013 (0.868, 1.182)	1.037 (0.925, 1.162)	1.477 (0.929, 2.348)
	Lag 02	1.054 (0.888, 1.252)	1.071 (0.944, 1.215)	1.300 (0.805, 2.099)

*CLRD* chronic lower respiratory disease, *PM2.5* particulate matter < 2.5 μm in diameter, *PM10* particulate matter < 10 μm in diameter

Italic ORs are statistically significant (P < 0.05). Measurement for every 10 μg/m^3^ increment of PM2.5, PM10, SO_2_, and O_3_ and 1 mg/m^3^ increment of CO

## Discussion

Our study’s results showed a decline in mortality from respiratory diseases and lung cancer in Shenyang, China. However, the respiratory and lung cancer death counts are continuing to increase with the aging of Shenyang’s population. The death count due to pneumonia has increased nearly threefold in 10 years. Our results suggest the effects of air pollution exposure on the number of deaths due to respiratory illness and lung cancer on subsequent days. We found a statistically significant increase in lung cancer mortality of 8.7% (95% CI, 0.8–17.2%) for a 10 μg/m^3^ increase in PM2.5; the increase was 10.4% (2.6–18.9%) in men for an average 10 μg/m^3^ increase in SO_2_ the previous day, and 13.2% (2–25.6%) for a 10 μg/m^3^ increase in SO_2_ at lag 02 days. For deaths due to respiratory diseases, the effect estimates were 4.7% (0–9.9%) for a 10 μg/m^3^ increase in PM2.5 on the same day, and 3.8% (1.3–6.3%) for a 1 mg/m^3^ increase of CO the previous day. The relationship between ozone exposure and respiratory mortality was also significant in people aged more than 65 years, with 10.2% (0.1–21.4%) increase in mortality from respiratory diseases.

Air pollution levels in China have been increasing rapidly. An analysis of data from the Global Burden of Diseases Study 2015 indicated that ambient PM2.5 accounted for 7.6% of total global deaths (about 4.2 million deaths), 59% of which being in East and South Asia (Cohen et al. 2017). Studies have investigated the short-term associations between daily increases in PM (PM2.5 and PM10) and mortality, especially in China (Tao et al. 2014; Xu et al. 2016; Yang et al. 2015). PM2.5 was ranked fifth in the risk factors of mortality in 2015 (Cohen et al. 2017). In this present study, we observed an elevated risk of dying from respiratory diseases associated with a high PM2.5 concentration at lag 1 day. Our findings also provided some evidence for an increased risk of lung cancer mortality caused by PM2.5 (Colao et al. 2016). A meta-analysis indicated that the meta-estimate for lung cancer mortality associated with PM2.5 was greater for males than for females (Huang et al. 2017). Similarly, our study demonstrated a positive relationship between PM2.5 and lung cancer mortality in males but not in females. PM2.5 mass concentration also showed increasing associations with both total respiratory-related mortality including lung cancer and lung cancer mortality only of the same day in the multiple-pollutant model. Our findings here corroborated the findings in previous studies (Crouse et al. 2015; Dominici et al. 2015).

Although ambient PM10 was significantly and positively associated with PM2.5 (Spearman’s correlation: *r* = 0.915, *P* < 0.01) and long-term exposure to elevated PM10 levels has been reported to generate a relative risk of all-cause and cause-specific mortality (Chen et al. 2016; Heinrich et al. 2013), significant positive correlation with respiratory diseases mortality risk was found only in population younger than 65 years with short-term exposure to PM10 in this study. Some studies in China obtained significant associations relating respiratory diseases risk to PM10; whereas, some studies conducted in city nearby Shenyang did not find the same association (Chen et al. 2010; Shang et al. 2013). Similar results were obtained in a study of 235,000 population, suggesting that expected short-term exposure to PM10 appears to have a limited impact on mortality (Carugno et al. 2014). Meanwhile, it has been reported that PM exposure tends to increase the natural death risk among people with chronic morbidity but has no significant risk among healthy persons (Alessandrini et al. 2016). RRs of lung cancer mortality were reported increases substantially among men in relation to long-term ambient concentrations of PM10 in a non-smoking cohort (Abbey et al. 1999). In our study, the mean PM10 concentration was not associated with lung cancer mortality in men or women. Increased OR was only observed in total population with every 10 μg/m^3^ increment of PM10 in the same day exposure. Since limited individual data were collected in this study, further research is needed to identify subgroups susceptible to elevated PM10 exposure.

Epidemiological studies indicated that daily changes in ambient concentrations of NO_2_ and SO_2_ trigger negative health effects on cardiopulmonary function, with both long-term and short-term exposure being associated with mortality risk (Brunekreef et al. 2009; Ghozikali et al. 2015; Int Panis et al. 2017; Miri et al. 2016). Our study found a relationship between lung cancer mortality and elevated SO_2_ level. Diurnal average SO_2_ concentrations with 2 or 3 previous-day exposure was associated with an increased lung cancer mortality risk, especially in males. Furthermore, single-day effects of the previous day (lag 1) of PM2.5, PM10, SO_2_, NO_2_, and CO, or the moving averages over the same day and previous day (lag 01) of PM2.5 and SO_2_, exposure substantially increased the risk of respiratory diseases mortality in the population younger than 65 years. NO_2_ and SO_2_ are often considered as indicators of traffic-related air pollution; the harmful effects of NO_2_ and SO_2_ would last with the increase in traffic intensity. Elevated risk of total respiratory-related mortality was also observed for SO_2_ (lag 01 and lag 02) when adjusted for the other pollutants. Results from a time series study (Zhang et al. 2015) confirmed that short-term exposure to SO_2_ was associated with 1.34% increases in respiratory disease emergency admissions in Beijing. However, in our study, no significant (*P* < 0.05) effects of SO_2_ were found for any respiratory disease deaths or lung cancer deaths in the multiple-pollutant model, except for the overall deaths combining respiratory diseases and lung cancer. Also, both respiratory disease deaths and lung cancer deaths had no significant association with exposure to NO_2_ in the single-pollutant model, but for population aged over 65 years, NO_2_ on lag 02 days seem to decline the daily mortality of respiratory disease. Inexplicably, in the multiple-pollutant model the average concentration of lag days for NO_2_ was associated with lower risk of respiratory disease deaths. The effect may be due in part to the potential for exposure misclassification, residual confounding, other unmeasured component of traffic pollution, and co-pollutant effects between NO_2_ and other pollutants (Hesterberg et al. 2009). Since lack of suitable evidence for personal exposure, the health effect that could result from exposure to the combination of the pollutants rather than individual pollutants could not be determined based on our data. It has been proved that ambient concentrations were not associated with their corresponding personal exposures for gaseous pollutants (Sarnat et al. 2001). Measured information of ambient air pollutants concentrations in more detail with the hour-to-hour variations rather than 24-h integrated were needed to describe the health risk associated with human exposure to air pollutants.

The results of this study also suggested that the mortality rate of respiratory diseases has direct significant correlations with CO concentrations in the air, in both older and younger populations. The acute effects of CO exposure occur with a 1-day lag. Similar studies have reported that increment concentrations of urban CO were related to respiratory mortality, especially during the warmer months (Atkinson et al. 2016). In the present study, about 85.95 and 91.86% of respiratory deaths were observed in males and females aged ≥ 65 years, respectively. Elderly persons in Shenyang may be at high risk for respiratory diseases because of the long-term exposure to industrial dust at their early life and the short-term exposure to air pollutants. As a traffic-related air pollutant, CO requires ample attention for its health impacts. Gender-stratified analyses were also performed to calculate the ORs of mortality associated with CO; a significant mortality impact attributable to CO was found in men, and only a low marginal effect (95% CI, 0.997–1.07) was found in women. Several epidemiological studies have shown that men were more susceptible to CO levels (Qorbani et al. 2012) or other air pollutant exposure than women (Xu et al. 2016). Whether mask usage is the key reason for females in Shenyang to be less susceptible to the harmful effects of CO remains unclear. Thus, further studies that assess exposure to the individual level are necessary.

Short-term ozone exposure has been reported to be associated with transient decrements in lung functions and increased respiratory symptoms (Chen et al. 2017; Ito et al. 2005). There is growing evidence that adverse effects of ozone exposure induce increased mortality risks in the aging population (Chen et al. 2017; Nuvolone et al. 2017). Our findings suggest that ozone exposure could lead to more deaths from respiratory diseases in people aged ≥ 65 years in Shenyang. However, the ozone-mortality association was not significant in the younger population. We found ozone daily concentration was negatively related with the other pollutants. Ozone is mainly produced from its major anthropogenic precursors such as the nitrogen oxides (NO_x_) and volatile organic compounds (VOCs). Previous study suggested that emission changes in VOCs might have played a more important role in the observed increase of surface ozone (Ma et al. 2016). The variation of reactivates of VOCs in morning traffic rush time at weekend or weekday may consequently change the concentration of surface ozone (Qin et al. 2004). The correlation between daily respiratory death and ozone were also different in winter and summer (Lindgren et al. 2009). To further understand the effect of ozone contributing to respiratory mortality, more detailed data and analysis were necessary.

The hazards of pneumoconiosis, a systemic occupational disease caused by long-term dust inhalation, are still serious in the old industrial city of Shenyang. Total of 289 individuals died from pneumoconiosis in the period of 2005–2015 in Shenyang. Pneumoconiosis is generally caused by long-term inhalation of dust (CDC 2012). So far, there is no evidence from previously published information in the association between mortality of pneumoconiosis and ambient air pollutants. We found every 10 μg/m^3^ of lag 1 day O_3_ concentration result a 50.5% increases of pneumoconiosis death and a confusing results that PM2.5, PM10, and CO show a small response shift effect to pneumoconiosis. It is unclear whether the association is due to a few outliers that occurred since every day death sample sizes were small or is attributable to confounding by some individual unknown or unmeasured factors. Most of the pneumoconiosis cases in this study were aged 80 and over. Additional analyses by cause of death are needed to examine the causal association between excess mortality and exposure to air pollution.

Multi-pollutant models which include terms of estimated population exposure for several pollutants were commonly used to identify the pollutant responsible for the observed effects (Vedal and Kaufman 2011). Multi-pollutant approach had been applied to examine the effect of multi-pollutant mixtures (Dominici et al. 2010). In this study, the main three pollutants of PM2.5, SO_2_, and NO_2_ were introduced into multiple-pollutant models to examine the independence of any associations observed in single-pollutant models. Due to the presence of highly collinear components among the ambient concentrations of air pollutant, it is important to conduct the multi-pollutant models to examine the role of these pollutants in multi-pollutant models rather than single-pollutant model. And we found that increase in the number of overall respiratory deaths were related with every 10 μg/m^3^ increases in the same day ambient concentrations of PM2.5 or the mean concentrations of SO_2_ of the same day and the previous 1 or 2 days.

This study has several limitations. A primary limitation of this study, and other similar studies in this field, is that air pollution exposure in the population is not assessed at the individual level, which may lead to aggregation bias. Furthermore, personal behaviors such as breathing mask usage and amount of time spent outdoors may also affect personal exposures. Females are more likely to use masks than males, resulting in the underestimation of air pollution effects. Younger groups spend more time outdoors than the elderly, particularly in the winter; hence, the association with mortality may be overestimated. In addition, this study was conducted in a sub-humid temperate continental city with a long winter period, where people need 5 months of coal burning. Moreover, the specific location of Shenyang may also limit the generalizability of findings.

## Conclusions

In this study, we have found positive associations between daily concentrations of air pollutants and mortality from respiratory diseases and lung cancer in Shenyang, China. We conclude that PM2.5, SO_2_, and CO exposures are significant risk factors for mortality from respiratory diseases and lung cancer in Shenyang, noting that younger people are more susceptible to the effects of particulate pollutants. PM2.5 and SO_2_ are also associated with increasing death counts due to lung cancer, especially in men. Our results confirm those of previous studies on possible acute adverse effects of air pollution exposure and further indicate which population subgroups are more susceptible to different air pollutants.

## Electronic supplementary material

Supplementary Table 1 (DOC 68 kb)
