# Supplementary material for: Temporal trends in respiratory mortality and short-term effects of air pollutants in Shenyang, China
Source: Environ Sci Pollut Res Int. 2018 Feb 9;25(12):11468–79. doi: 10.1007/s11356-018-1270-5 (PMC5940718; doi:10.1007/s11356-018-1270-5)
Supplement: Supplementary file 1 — (DOC 68 kb) [file 11356_2018_1270_MOESM1_ESM.doc]

Supplementary Table 1. Effect estimates of air pollutants on daily mortality of lung cancer and respiratory diseases in different population groups using single-pollutant models

|  |  | <65 years old | | ≥65 years old | | Male | | Female | |
| --- | --- | --- | --- | --- | --- | --- | --- | --- | --- |
| - |  | Lung cancer | Respiratory diseases | Lung cancer | Respiratory diseases | Lung cancer | Respiratory diseases | Lung cancer | Respiratory diseases |
| PM2.5 | Lag0 | 1.084(0.995,1.181) | 1.107(0.965,1.271) | 1.057(0.995,1.123) | 1.019(0.970,1.071) | **1.077(1.008,1.150)** | 1.011(0.949,1.076) | 1.047(0.969,1.130) | 1.052(0.981,1.127) |
|  | Lag1 | 1.030(0.945,1.123) | **1.303(1.137,1.493)** | 1.021(0.962,1.085) | 1.019(0.969,1.072) | 1.043(0.976,1.114) | 1.042(0.978,1.110) | 0.995(0.922,1.075) | 1.055(0.983,1.132) |
|  | Lag2 | 1.015(0.931,1.108) | 0.981(0.855,1.125) | 1.023(0.963,1.087) | 0.996(0.947,1.047) | 1.030(0.964,1.101) | 0.961(0.902,1.023) | 1.007(0.931,1.088) | 1.042(0.971,1.118) |
|  | Lag01 | 1.075(0.969,1.192) | **1.239(1.048,1.464)** | 1.066(0.991,1.146) | 1.004(0.946,1.067) | 1.074(0.992,1.163) | 1.021(0.946,1.101) | 1.059(0.965,1.163) | 1.037(0.953,1.128) |
|  | Lag02 | 1.061(0.945,1.191) | 1.149(0.951,1.387) | 1.054(0.973,1.143) | 0.986(0.922,1.055) | 1.057(0.968,1.154) | 0.981(0.902,1.068) | 1.055(0.951,1.171) | 1.031(0.939,1.133) |
| PM10 | Lag0 | 1.104(0.998,1.223) | 1.087(0.922,1.282) | 1.059(0.984,1.139) | 1.020(0.961,1.083) | 1.080(0.998,1.169) | 0.986(0.914,1.064) | 1.062(0.967,1.165) | 1.083(0.997,1.177) |
|  | Lag1 | 1.002(0.903,1.112) | **1.284(1.090,1.511)** | 1.020(0.948,1.096) | 1.013(0.953,1.076) | 1.018(0.940,1.102) | 1.029(0.954,1.111) | 1.009(0.920,1.107) | 1.055(0.969,1.148) |
|  | Lag2 | 0.989(0.891,1.098) | 0.985(0.833,1.166) | 1.021(0.949,1.099) | 0.993(0.935,1.055) | 1.023(0.944,1.108) | 0.949(0.880,1.024) | 0.994(0.904,1.092) | 1.055(0.969,1.148) |
|  | Lag01 | 1.077(0.950,1.220) | 1.216(0.994,1.487) | 1.072(0.982,1.171) | 1.010(0.939,1.087) | 1.058(0.960,1.165) | 1.003(0.914,1.10) | 1.099(0.982,1.230) | 1.069(0.965,1.183) |
|  | Lag02 | 1.052(0.915,1.210) | 1.170(0.930,1.472) | 1.064(0.965,1.173) | 1.000(0.922,1.086) | 1.054(0.947,1.172) | 0.978(0.882,1.085) | 1.071(0.945,1.215) | 1.070(0.955,1.200) |
| SO_2_ | Lag0 | 1.086(0.987,1.196) | 1.084(0.929,1.264) | 1.020(0.954,1.090) | 1.021(0.966,1.079) | 1.065(0.99,1.146) | 1.010(0.942,1.083) | 1.000(0.919,1.089) | 1.051(0.973,1.136) |
|  | Lag1 | 1.050(0.954,1.155) | **1.359(1.168,1.581)** | 1.053(0.985,1.125) | 1.019(0.963,1.077) | **1.104(1.026,1.189)** | 1.054(0.982,1.131) | 0.974(0.895,1.060) | 1.052(0.973,1.137) |
|  | Lag2 | 0.971(0.883,1.068) | 0.924(0.793,1.076) | 1.050(0.982,1.123) | 0.989(0.935,1.046) | 1.042(0.968,1.122) | 0.957(0.893,1.026) | 0.997(0.914,1.087) | 1.016(0.939,1.098) |
|  | Lag01 | 1.098(0.976,1.234) | **1.335(1.107,1.609)** | 1.074(0.990,1.166) | 1.006(0.940,1.077) | **1.130(1.033,1.237)** | 1.041(0.954,1.135) | 1.007(0.907,1.118) | 1.037(0.943,1.140) |
|  | Lag02 | 1.044(0.912,1.195) | 1.230(0.988,1.533) | 1.098(0.999,1.208) | 1.001(0.925,1.084) | **1.132(1.020,1.256)** | 1.019(0.921,1.127) | 1.004(0.889,1.135) | 1.032(0.924,1.152) |
| NO_2_ | Lag0 | 1.074(0.933,1.237) | 0.989(0.789,1.238) | 1.036(0.938,1.143) | 0.992(0.915,1.075) | 1.075(0.964,1.198) | 0.96(0.867,1.063) | 1.006(0.888,1.141) | 1.034(0.924,1.157) |
|  | Lag1 | 1.056(0.919,1.215) | **1.403(1.129,1.743)** | 1.047(0.949,1.155) | 0.981(0.904,1.064) | 1.107(0.993,1.233) | 1.027(0.926,1.138) | 0.964(0.851,1.093) | 1.017(0.909,1.139) |
|  | Lag2 | 0.997(0.867,1.147) | 0.992(0.794,1.238) | 1.043(0.944,1.152) | 0.924(0.851,1.003) | 1.052(0.944,1.173) | 0.908(0.819,1.006) | 0.992(0.873,1.126) | 0.963(0.859,1.079) |
|  | Lag01 | 1.097(0.920,1.307) | 1.272(0.963,1.68) | 1.072(0.948,1.211) | 0.935(0.846,1.034) | 1.132(0.990,1.294) | 0.959(0.844,1.090) | 1.002(0.856,1.172) | 0.980(0.852,1.127) |
|  | Lag02 | 1.086(0.888,1.328) | 1.241(0.898,1.715) | 1.079(0.936,1.244) | **0.873(0.776,0.981)** | 1.125(0.963,1.313) | 0.897(0.773,1.042) | 1.016(0.846,1.220) | 0.922(0.783,1.085) |
| CO | Lag0 | 1.033(0.99,1.078) | 1.007(0.942,1.076) | 1.012(0.983,1.042) | 1.022(0.997,1.048) | 1.019(0.986,1.052) | 1.016(0.985,1.047) | 1.018(0.981,1.056) | 1.027(0.992,1.064) |
|  | Lag1 | 1.018(0.976,1.062) | **1.135(1.055,1.22)** | 0.999(0.971,1.028) | **1.026(1.001,1.053)** | 1.01(0.979,1.043) | **1.041(1.009,1.075)** | 0.996(0.959,1.033) | 1.033(0.997,1.07) |
|  | Lag2 | 0.997(0.956,1.041) | 0.988(0.926,1.054) | 1.013(0.984,1.043) | 0.996(0.972,1.02) | 1.009(0.978,1.042) | 0.989(0.959,1.018) | 1.006(0.968,1.046) | 1.005(0.971,1.04) |
|  | Lag01 | 1.027(0.955,1.104) | 1.097(0.966,1.247) | 1.027(0.977,1.08) | 1.02(0.977,1.065) | 1.026(0.971,1.085) | 1.03(0.976,1.087) | 1.028(0.964,1.096) | 1.027(0.965,1.092) |
|  | Lag02 | 0.999(0.905,1.101) | 0.994(0.848,1.164) | 1.033(0.964,1.107) | 0.986(0.932,1.043) | 1.013(0.939,1.093) | 0.984(0.917,1.056) | 1.037(0.95,1.132) | 0.991(0.915,1.073) |
| O_3_ | Lag0 | 1.032(0.904,1.179) | 1.171(0.945,1.452) | 1.005(0.916,1.103) | 1.035(0.959,1.117) | 0.998(0.902,1.105) | 1.058(0.96,1.164) | 1.037(0.921,1.168) | 1.038(0.933,1.156) |
|  | Lag1 | 1.013(0.883,1.161) | 0.860(0.698,1.060) | 0.954(0.870,1.046) | 1.048(0.971,1.132) | 0.957(0.863,1.060) | 1.025(0.931,1.129) | 0.994(0.882,1.120) | 1.028(0.924,1.144) |
|  | Lag2 | 0.948(0.829,1.083) | 0.984(0.790,1.227) | 1.011(0.921,1.110) | 1.074(0.994,1.161) | 0.948(0.855,1.050) | 1.059(0.961,1.168) | 1.066(0.946,1.200) | 1.069(0.959,1.192) |
|  | Lag01 | 1.036(0.889,1.208) | 1.034(0.812,1.318) | 0.979(0.881,1.089) | 1.063(0.974,1.160) | 0.981(0.874,1.102) | 1.080(0.968,1.205) | 1.018(0.888,1.168) | 1.034(0.915,1.168) |
|  | Lag02 | 1.014(0.856,1.201) | 1.004(0.767,1.313) | 0.992(0.882,1.116) | **1.102(1.001,1.214)** | 0.977(0.859,1.111) | 1.114(0.986,1.258) | 1.033(0.887,1.203) | 1.063(0.928,1.216) |

Bold ORs are statistically significant (P < 0.05)
